# Carbon/nitrogen metabolism and stress response networks – calcium-dependent protein kinases as the missing link?

**DOI:** 10.1093/jxb/erab136

**Published:** 2021-03-31

**Authors:** Hugo L S Alves, Cleverson C Matiolli, Rafael C Soares, M Cecília Almadanim, M Margarida Oliveira, Isabel A Abreu

**Affiliations:** 1 Instituto de Tecnologia Química e Biológica António Xavier, Universidade Nova de Lisboa (ITQB NOVA), Avenida da República, 2780-157 Oeiras, Portugal; 2 University of Illinois, USA

**Keywords:** Calcium-dependent protein kinase, CDPK, Ca^2+^, carbon metabolism, growth, nitrogen metabolism, SnRK1, stress, TOR

## Abstract

Calcium-dependent protein kinases (CDPKs) play essential roles in plant development and stress responses. CDPKs have a conserved kinase domain, followed by an auto-inhibitory junction connected to the calmodulin-like domain that binds Ca^2+^. These structural features allow CDPKs to decode the dynamic changes in cytoplasmic Ca^2+^ concentrations triggered by hormones and by biotic and abiotic stresses. In response to these signals, CDPKs phosphorylate downstream protein targets to regulate growth and stress responses according to the environmental and developmental circumstances. The latest advances in our understanding of the metabolic, transcriptional, and protein–protein interaction networks involving CDPKs suggest that they have a direct influence on plant carbon/nitrogen (C/N) balance. In this review, we discuss how CDPKs could be key signaling nodes connecting stress responses with metabolic homeostasis, and acting together with the sugar and nutrient signaling hubs SnRK1, HXK1, and TOR to improve plant fitness.

## Teaser

This review discusses the possible links between Ca^2+^ signaling through calcium-dependent protein kinases and the regulation of carbon and nitrogen metabolism during stress.

## Introduction

Plants colonized the land around 480–360 million years ago (Kenrick and Crane, 1997). In contrast to the aquatic environment, organisms living in a terrestrial habitat need to be able to endure desiccation and significant oscillations in temperature. The astonishing array of strategies deployed by plants to overcome these challenges reflects the diversification and expansion of species and, at the molecular level, this relied on the multiplication of signaling pathways and gene families (Corrêa *et al.*, 2008; Hamel *et al.*, 2014; Valmonte *et al.*, 2014) (Box 1). Furthermore, the sessile nature of plants also requires prompt and effective stress-counteracting measures to ensure survival in an ever-changing environment. Plants perceive changes in surrounding physical and chemical parameters through cellular receptors, which in turn trigger physiological and morphological changes to adapt to the environmental circumstances (Lamers *et al.*, 2020). All these changes usually start with a chain reaction of post-translational modifications (PTMs) triggered by perception of the stress signals, which transduces the signals into appropriate physiological responses. Among PTMs, protein phosphorylation plays significant roles in changing the operation of cellular activities in response to stress and metabolic demands (Baena-González *et al.*, 2007; Xiong *et al.*, 2013; Liu *et al.*, 2017). The covalent attachment of a phosphate moiety changes the conformation of the target protein, modulating its protein–protein interactions, activity, subcellular location, or stability (Ormancey *et al.*, 2017). PTM is the regulation of choice in the plant arsenal of perception and signaling mechanisms, allowing the organism to adapt to changes in environmental parameters quickly.

Box 1. **Diversification of CDPKs in land plants: the map to conquer new territories**

CDPKs exist in the Alveolata (ciliates and apicomplexans) and in the Archaeplastida, which consists of the red algae SCRP clade (Stylonematophyceae, Compsopogonophyceae, Rhodellophyceae, Porphyridiophyceae), green algae, and land plants (Billker *et al.*, 2009; Hamel *et al.*, 2014; Valmonte *et al.*, 2014). Intron conservation between protist and plant CDPKs suggests that these proteins share a common ancestor (Harper *et al.*, 1991; Zhang and Choi, 2001). Nevertheless, it remains an open question as to whether the presence of CDPKs in these two evolutionary groups resulted from a horizontal transfer or if they appeared before the separation of the Alveolata and Archaeplastida branches.

Genome-wide analysis of CDPKs from algae to higher plants has shed light on CDPK gene evolution in the Viridiplantae, and over 500 CDPKs have been identified among 19 plant species. The number of CDPK genes in each plant genome probably reflects the evolutionary events of large genome duplications and polyploidization (Valmonte *et al.*, 2014), and examination of the chromosomal distribution of CDPK loci suggests that family expansion has resulted mainly from large DNA rearrangements (Hamel *et al.*, 2014). CDPKs have diversified into ~10 genes in green algae, 10–20 genes among lower land plants (such as Bryophyta, Pteridophyta, Gymnospermae), and ~30–40 genes in angiosperms (monocots and dicots), with Arabidopsis and rice displaying 34 and 30 CDPKs, respectively (Asano *et al.*, 2005; Hamel *et al.*, 2014). Remarkably, the expansion of the CDPK family seems to correlate with the increased complexity of plant architecture and the exposure to new types of stress—such as drought and extreme temperature changes—which are associated with the progression of land occupation.

There is a clear diversification of CDPKs into four major evolutionary groups (I–IV) in plants that conquered the land, namely Bryophyta, Pteridophytes, Gymnospermae, and Angiospermae (Asano *et al.*, 2005; Hamel *et al.*, 2014; Valmonte *et al.*, 2014). Furthermore, the timing of this diversification together with the functional relevance of CDPKs in conveying external signals suggests their putative role in the transition or adaptation of plant life to terrestrial environments. The challenges imposed by life outside the aquatic environment involved CDPKs in more complex physiological, developmental, and stress-response networks. Hence, more in-depth knowledge of the evolution, structure, and function of CDPKs might inform possible future strategies to help to overcome environmental constraints to crop performance.

Over the last century, the divalent calcium cation (Ca^2+^) has been recognized as a pivotal second messenger in eukaryotic cells (Dodd *et al.*, 2010; Kudla *et al.*, 2018). The perception of environmental and developmental cues triggers rapid transient changes in cytosolic Ca^2+^ concentrations ([Ca^2+^]_cyt_), generating spatio-temporal wave patterns that carry stimulus-specific information known as ‘Ca^2+^ signatures’ (Behera *et al.*, 2018; Ma *et al.*, 2019; Gjetting *et al.*, 2020). By reading and decoding these signals, plants trigger specific responses to cope with the stress or to make developmental decisions. For instance, cold temperatures trigger a rapid increase in [Ca^2+^]_cyt_ through the activation of Ca^2+^ channels (Ma *et al.*, 2015). Salt and drought stress signaling also rely on generating [Ca^2+^]_cyt_ spikes to elicit physiological responses (Kiegle *et al.*, 2000; Jiang *et al.*, 2019). Molecular signatures of pathogens are detected by pattern recognition receptors (PRRs), triggering a burst of reactive oxygen species (ROS), secondary metabolite production, and Ca^2+^ influx into the cytosol (Maintz *et al.*, 2014). Ca^2+^-binding proteins, such as calcium-dependent protein kinases (CDPKs), read Ca^2+^ signatures through their Ca^2+^-binding domains and translate the encoded information into phosphorylation of downstream targets, such as enzymes or transcription factors (Zhu *et al.*, 2007; Almadanim *et al.*, 2017; for detailed information see (Dodd *et al.*, 2010; Batistič and Kudla, 2012; Yip Delormel and Boudsocq, 2019). Most CDPKs have predicted N-myristoylation and N-palmitoylation sites, which anchor them to cellular membranes and determine the subset of protein targets with which they interact (Martín and Busconi, 2000; Asai *et al.*, 2013; Simeunovic *et al.*, 2016; Saito *et al.*, 2018). The subcellular localization, distribution within the organism, and timing of expression of CDPKs might all contribute to the proper decoding of individual Ca^2+^ signatures, determining distinct spatial-temporal transduction outcomes of the Ca^2+^ signals. There have been recent comprehensive reviews about the properties and functions of CDPKs in Arabidopsis (Yip Delormel and Boudsocq, 2019), their phylogenetic and functional relationships in green plants (Hamel *et al.*, 2014; Valmonte *et al.*, 2014), and their roles in signaling during abiotic and biotic stress (Boudsocq and Sheen, 2013).

Carbon and nitrogen metabolism are intimately linked to growth and stress responses, and tight control of their fluxes in cellular metabolism and throughout the plant is essential to ensure survival and reproduction under environmental constraints (Margalha *et al.*, 2019). The disruption of metabolic homeostasis is sensed by the conserved kinases SUCROSE NON-FERMENTING RELATED KINASE1 (SnRK1), TARGET OF RAPAMYCIN (TOR), and HEXOKINASE1 (HXK1), which are responsible for setting cellular processes according to metabolic demands and cellular activities (Baena-González *et al.*, 2007; Xiong *et al.*, 2013; Dobrenel *et al.*, 2016). Briefly, SnRK1 perceives cellular energy deficiency and triggers changes in plant metabolism and transcription to repress anabolism and promote catabolism (Baena-González *et al.*, 2007). Conversely, an abundance of energy and nutrients (e.g. glucose and nitrogen availability) activates TOR kinase, which in turn stimulates protein translation and growth (Xiong *et al.*, 2013). The SnRK1–TOR regulatory module also integrates hormone and environmental stress signals to modulate growth. The plant HXK1 possess independent catalytic (i.e. hexose phosphorylation to enter glycolysis) and glucose-sensing activities. The perception of hexoses by HXK1 influences several hormone signaling pathways, affecting plant development and stress responses (Li and Sheen, 2016).

It is largely accepted that plant signaling pathways integrate parallel signals that convey environmental and metabolic information (Margalha *et al.*, 2019); however, we still lack a comprehensive picture of the molecular mechanisms implicated in this multi-signal processing. In this review, we consider how CDPKs integrate information from environmental and internal cues to regulate plant metabolism. Accumulating evidence provides a framework for evaluating the role of these Ca^2+^ relay proteins in energy homeostasis and carbon/nitrogen (C/N) balance.

## Connecting the dots: CDPKs as nodes of development, metabolic, and stress signaling

The adaptation of a plant to the environment is mediated by an extensive set of cellular receptors and their respective downstream signaling pathways that convey hormonal and environmental cues. Hormone perception, changes in sugar concentrations, and the onset of stress can result in dynamic [Ca^2+^]_cyt_. In this context, CDPKs act as important hubs in signal processing and integration: multiple stress signals converge into a single CDPK, and different CDPKs can phosphorylate the same targets (Schulz *et al.*, 2013). Because plant hormone and sugar-signaling pathways interact at many levels to regulate plant development and stress responses (Ramon *et al.*, 2008; Li and Sheen, 2016; Wingler, 2018), we consider the question of how pervasive the involvement of CDPKs is within metabolic and hormone signaling pathways.

There are common phosphorylation targets between CDPKs and the abscisic acid (ABA)-responsive members of the SnRK2 family. For instance, SnRK2.6/OST1 (SUCROSE NON-FERMENTING RELATED PROTEIN KINASE2.6/OPEN STOMATA1) and CDPKs integrate stress-induced increases in [Ca^2+^]_cyt_ into downstream ABA-driven signal transduction pathways to control stomatal aperture. In this model, ABA enhances, or ‘primes’, the sensitivity of guard cells to increases in [Ca^2+^]_cyt_. The molecular mechanism involves the control of the phosphorylation of the S-type anion channel SLAC1 (SLOW ANION CHANNEL-ASSOCIATED1) by OST1, the CDPKs AtCPK3/6/21/23, and the PP2Cs PP2CA (PROTEIN PHOSPHATASE 2C A) and ABI1 (ABSCISIC ACID INSENSITIVE1) (Mori *et al.*, 2006; Geiger *et al.*, 2010; Brandt *et al.*, 2015). Interestingly, both AtCPK6 and OST1 are required for glucose-induced stomatal closure, suggesting that these kinases are at the crossroads of ABA and sugar signaling to regulate stomatal conductance (Li *et al.*, 2018). The chelating agent EGTA, which sequesters Ca^2+^ cations and dampens downstream Ca^2+^ signaling, impairs the stomatal closure in response to glucose, suggesting that AtCPK6—and maybe other CDPKs—is a key regulator of sugar signaling in guard cells. In addition, using the *HXK1*-knockout mutant, *glucose insensitive2-1* (*gin2-1*), Li *et al.* (2018) demonstrated that HXK1 is also necessary for the stomatal closure imposed by glucose treatment. However, it remains unknown whether the stomatal control mediated by HXK1 relies on its catalytic role in glucose catabolism—i.e. an entry point for glycolysis and the tricarboxylic acid (TCA) cycle—or on its glucose-sensing activity (Rolland *et al.*, 2006). *Zea mays* ZmCPK4, which is phylogenetically close to AtCPK6, also localizes in the plasma membrane, and its heterologous overexpression in Arabidopsis increases the ABA sensitivity of seed germination and stomatal closure (Jiang *et al.*, 2013). This finding raises the question as to whether the regulatory system of stomatal aperture observed in Arabidopsis involving CDPKs and SnRK2 is conserved between monocots and dicots.

Control of the transcriptional profile is also a hallmark of the interaction between sugars and ABA signaling, and there is circumstantial evidence for Ca^2+^ action at the interface between the sugar and ABA pathways. The transcriptional profiles associated with sugar and ABA treatments show a significant overlap (Li *et al.*, 2006), with both triggering transient changes in [Ca^2+^]_cyt_ that can affect global transcriptional activity, and the elevation of [Ca^2+^]_cyt_ induces the phosphorylation of critical ABA-responsive transcriptional regulators by CDPKs. For instance, AtCPK32 phosphorylates ABRE BINDING FACTOR4 (ABF4) *in vitro* (Choi *et al.*, 2005), and the two closely related Group I Arabidopsis CDPKs AtCPK4 and AtCPK11 are positive regulators of ABA signaling, both phosphorylating AtABF1 and AtABF4 *in vitro* (Zhu *et al.*, 2007). Importantly, overexpression of AtCPK32 affects ABA sensitivity (Choi *et al.*, 2005), highlighting the importance of CDPKs in water-stress responses. The notable fact that several ABA biosynthesis and signaling mutants also have altered sensitivity to sugars shows the need for further analysis of the role of Ca^2+^ in this ABA–sugar interaction.

Arabidopsis CDPKs and SnRK2 family members again interact to modulate ABA signaling, as seen before in the convergent regulation of stomatal dynamics by ABA and Ca^2+^. Several members of the SnRK2 family also phosphorylate AtABF1/4, among other ABFs, thus potentially integrating ABA and Ca^2+^ signals into specific transcriptional responses. Zhu *et al.* (2007) found that the protein levels of AtCPK4 and AtCPK11 transiently increase upon ABA treatment. Interestingly, disruption of AtCPK4 or AtCPK11 reduces seedling sensitivity to ABA but causes hypersensitivity to salt stress. It could be hypothesized that ABA ‘primes’ the responsiveness to [Ca^2+^]_cyt_ spikes by controlling the protein accumulation of these CDPKs, with persistence or repetition of stress potentially leading to specific Ca^2+^ signature patterns to adapt the plant’s physiology to cope with the stress. It would be interesting to investigate whether ABA influences AtCPK4/11 to modulate the dynamics of the [Ca^2+^]_cyt_ spikes that trigger the ‘Salt Overly Sensitive’ pathway that drives exclusion of Na^2+^ from cells during salt stress (Ji *et al.*, 2013).

Signal transduction through CDPKs seems to connect environmental stress signaling to growth and developmental control. Rice OsCPK7 transcripts are induced by gibberellins (GAs) and repressed by ABA and brassinolide, while, at the protein level, OsCPK7 is phosphorylated following GA treatment and imposition of cold stress (Abbasi *et al.*, 2004). A variety of hormones (e.g. ABA, cytokinin, and GA) and stresses induce transcripts of tobacco NtCPK1 (Yoon *et al.*, 1999), and it regulates GA homeostasis through phosphorylation of the basic leucine zipper (bZIP) transcription factor REPRESSION OF SHOOT GROWTH (RSG) in response to GA treatment (Ishida *et al.*, 2008). It remains to be determined whether NtCPK1 also regulates RSG activity and GA accumulation in response to other Ca^2+^ signals or if it only decodes GA-induced cellular changes in [Ca^2+^] to create a feedback loop to regulate GA homeostasis. Interestingly, NtCPK1 also phosphorylates the regulatory 26S proteasome subunit NtRpn3 to control cell fate (Lee *et al.*, 2003). Thus, NtCPK1 seems to be broadly involved in controlling plant architecture, thus potentially modulating plasticity in response to external and internal cues by regulating cell division, cell death, and GA signaling. It would be interesting to investigate possible connections between CDPK signaling and the glucose-induced regulation of the cell cycle mediated by TOR kinase (Xiong *et al.*, 2013).

In potato, StCPK3 phosphorylates StABF1 and StRSG1 *in vitro*, suggesting that regulation of GA homeostasis by CDPKs might be a conserved feature in plants. StCPK3 is proposed to be a key modulator of transcriptional processes at the onset of potato tuberization, as it can promote crosstalk between the GA and ABA signaling networks (Grandellis *et al.*, 2016). Furthermore, rice CDPKs mediate GA-driven changes in metabolic enzymes. Khan *et al.* (2005) have suggested that a 54-kDa rice CDPK can phosphorylate the GLYCERALDEHYDE-3-PHOSPHATE DEHYDROGENASE (GAPDH) and MALATE DEHYDROGENASE (MDH) enzymes upon treatment with GA_3_, but further validation is necessary to prove that there is direct phosphorylation of these metabolic enzymes by a CDPK. Another interesting example connecting GA signaling and carbon metabolism is the induction by sugar starvation of *OsCPK7*, which results in negative regulation of GA biosynthetic genes, (Ho *et al.*, 2013). OsCPK7 modulates plant growth, seed yield, and seed quality, the latter through regulation of seed amylose content (Ho *et al.*, 2013; Jiang *et al.*, 2018).

The regulation of CDPK target proteins through phosphorylation might require the action of 14-3-3 proteins, which bind to phosphopeptide motifs and modulate protein stability, activity, interactions, and localization (Camoni *et al.*, 1998; Ormancey *et al.*, 2017). In a series of elegant experiments, Ito *et al.* (2014) demonstrated that, after Ca^2+^-induced activation, NtCPK1 autophosphorylates and binds to a 14-3-3 protein and RSG, forming a scaffold. Subsequently, NtCPK1 phosphorylates both the protein and RSG, promoting the transfer of 14-3-3 to RSG. This mechanism retains the RSG in the cytoplasm, thus hindering its binding to the *NtGA20ox1* promoter to regulate GA homeostasis. These findings provide a mechanistic link between changes in [Ca^2+^]_cyt_ and transcription regulation and modulation of hormone homeostasis, which affect plant growth and stress responses. In rice, OsCPK21 phosphorylates the 14-3-3 protein OsGF14e to regulate genes responsive to ABA and salt stress (Chen *et al.*, 2017). OsGF14e also plays a role in biotic responses because it is a positive regulator of resistance to *Magnaporthe oryzae* (blast) infection (Liu *et al.*, 2016), but the involvement of OsCPK21 in biotic stress responses remains to be investigated. In addition, the expression of another 14-3-3 protein, OsGF14c, is induced by overexpression of *OsCPK7* (Ho *et al.*, 2013). The association between CDPKs and 14-3-3 proteins seems to coordinate a wide array of plant responses, connecting developmental and stress signaling pathways (Ormancey *et al.*, 2017).

Biotic stress has a significant impact on plant resources because pathogens hijack nutrients from plants, which in turn synthesize secondary metabolites to fight the invading organism or to eliminate the affected tissues or organs. In this context, CDPKs also modulate defense mechanisms upon the perception of Ca^2+^ influx triggered by the detection of pathogen effectors (effector triggered immunity, ETI). The Arabidopsis CDPKs AtCPK4/5/6/11 phosphorylate the transcription factors WRKY8/28/48 to modulate the expression of downstream biotic-responsive genes (Ishihama and Yoshioka, 2012; Gao *et al.*, 2013). Cui *et al.* (2020) have recently demonstrated that the rapeseed CDPKs BnaCPK5/6/11 phosphorylate the WRKY transcription factor BnaWSR1 to regulate the accumulation of salicylic acid (SA) and leaf senescence. In Arabidopsis, the regulation of camalexin biosynthesis in response to fungal pathogens is mediated by both CDPKs and MAPKs. The pathogen-responsive CDPKs AtCPK5 and AtCPK6 phosphorylate the DNA-binding domain of WRKY33 and increase its binding to DNA, while MPK3 and MPK6 phosphorylate the WRKK33 N-terminal region to enhance its transactivation activity (Zhou *et al.*, 2020).

Interestingly, OsCPK7 transcripts, which are induced by sugar starvation, are also induced by jasmonic acid (JA), SA, and by *Xanthomonas oryzae* pv. *oryzae* (*Xoo*). Overexpression of *OsCPK7* up-regulates the rice genes *PATHOGEN RELATED1/4/10a* (*PR1*, *PR4*, and *PR10a*), acting as a positive regulator of resistance to *Xoo* infection (He *et al.*, 2018). These findings raise the interestingly possibility that *OsCPK7* could integrate sugar status with plant defense. The promoter element responsible for *OsPR10a* transcriptional induction is a W-box (Hwang *et al.*, 2008) and this is known to bind to WRKY transcription factors, which are biotic stress-response modulators. It is possible that OsCPK7 regulates WRKY DNA binding in a similar manner to AtCPK3/6 and WRKY33 in Arabidopsis, but this assumption is yet to be demonstrated. Overall, it is clear that CDPKs can convert Ca^2+^ signals in the modulation of WKRY-mediated transcriptional activity, conferring on them a central place in plant biotic stress responses and crosstalk with sugar signaling.

Finally, many sugar-insensitive mutants are allelic to ABA and ethylene signaling components, and recent work has shown that ABA modulates the activity of the energy sensor SnRK1 (Belda-Palazón *et al.*, 2020) and the nutrient sensor TOR (Wang *et al.*, 2018). The ABA-response regulator SnRK2 phosphorylates the TOR regulatory subunit RaptorB, disrupting its interaction with TOR and attenuating the growth-promoting activity of the TOR complex (Wang *et al.*, 2018). This study also demonstrated that TOR reduces ABA sensitivity by phosphorylating the ABA sensors PYRABACTIN RESISTANCE LIKE1 and 4 (PYL1/4), dampening the action of SnRK2s such as OST1. As discussed above, previous evidence suggests that OST1 and AtCPK3/6/21/23 act in concert to integrate ABA and Ca^2+^ signals to control stomatal aperture. It would be interesting to determine whether SnRK2 and CDPKs also interact to regulate growth through modulation of the activity of the TOR complex.

## Energy and nutrient homeostasis: a matter of C/N balance

An early clue for the existence of crosstalk between carbohydrates and CDPK-mediated Ca^2+^ signaling to modulate primary metabolism came from Ohto and Nakamura (1995). They identified a putative tobacco membrane-bound CDPK with increased autophosphorylation activity upon sugar treatment of the leaves, which mediates the sugar-induced expression of β-amylase and sporamin (Iwata *et al.*, 1998). Interestingly, the activation of CDPK is more evident in younger leaves, suggesting that it could be important for metabolic homeostasis in growing tissues and organs. Later, Asano *et al.* (2002) showed that OsCPK23 phosphorylates a serine residue in the N-terminal region of sucrose synthase (SUSY) and activates it, which plays a role in sucrose unloading from the phloem to developing seeds. Down-regulation of OsCPK23 by antisense RNA results in low SUSY activity and watery seeds that contain high sucrose content and have reduced starch accumulation. In addition, the rubber tree HbCDPK1 has been suggested to regulate sucrose metabolism during ethephon-stimulated latex production (Zhu *et al.*, 2010).

The regulation of central carbon metabolism is crucial for plant fitness in changing environments. Carbon partitioning during photosynthesis is largely controlled by FRUCTOSE-2,6-BISPHOSPHATASE (F2KP), which is a bifunctional enzyme that catalyses the interconversion Fru-6-P ↔ Fru-2,6-P_2_ and has a central role in metabolism by regulating carbohydrate partitioning between sucrose and starch (Draborg *et al.*, 2001; Nielsen *et al.*, 2004; Villadsen *et al.*, 2005). The ratio of Fru-6-P/Fru-2,6-P_2_ is regulated by water stress in barley and by light conditions in rice leaves (Ramachandra Reddy and Das, 1987; Villadsen *et al.*, 2005), suggesting that F2KP activity is modulated by both environmental stress and photosynthesis. For instance, increased levels of Fru-2,6-P_2_ direct photosynthetic sugars to starch synthesis (Scott *et al.*, 2000; Villadsen *et al.*, 2005). Interestingly, SnRK1 and AtCPK3 converge in the phosphorylation of the Ser303 residue of AtF2KP (Kulma *et al.*, 2004; Nukarinen *et al.*, 2016), suggesting that AtF2KP could be a hub of metabolic and environmental convergent signals that require adjustments within central carbon metabolism.

**Fig. 1.**
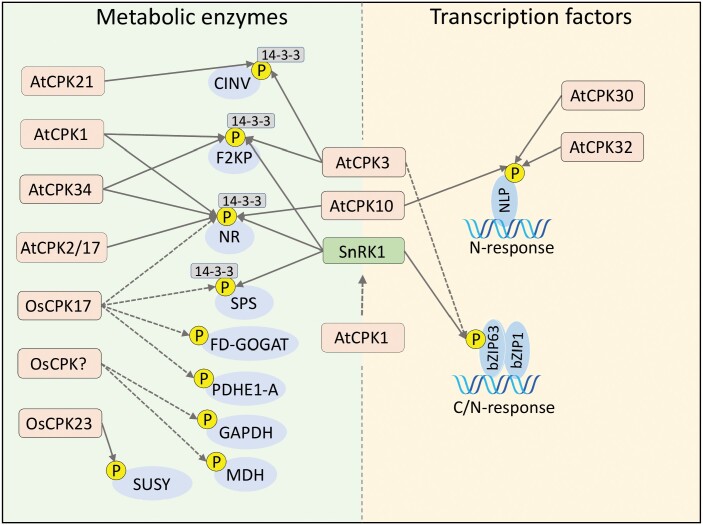
Integration of C/N metabolism by calcium-dependent protein kinases (CDPKs) and the sugar/energy sensor SUCROSE NON-FERMENTING RELATED KINASE1 (SnRK1). The C/N-related enzymes and transcriptions factors commonly targeted by CDPKS and SnRK1 are shown. Phosphorylation targets of CDPKs (orange) and SnRK1 (green) are shown in blue, and 14-3-3 phospho-binding proteins are shown in gray. Solid arrows indicate direct phosphorylation (*in vivo* or *in vitro*) and dashed arrows represent putative direct or indirect modulation.

The Arabidopsis CDPKs AtCPK1, AtCPK10, and AtCPK34 phosphorylate both F2KP and nitrate reductase (NR) peptides *in vitro* (Curran *et al.*, 2011). AtCPK1 is a positive regulator of salt and drought tolerance (Huang *et al.*, 2018), while AtCPK10 is involved in ABA signaling and the response to drought stress (Zou *et al.*, 2010), and AtCPK34 regulates pollen-tube growth (Myers *et al.*, 2009). In addition, AtCPK3 and AtCPK21 phosphorylate Arabidopsis CYTOSOLIC/PLASTIDIAL MITOCHONDRIAL INVERTASE 1 (AtCINV1) to promote 14-3-3 protein binding and increased activity (Gao *et al.*, 2014). The CINV1 invertases mediate sucrose degradation into fructose and glucose, and the CDPK-mediated phosphorylation of AtCINV1 might occur in coordination with the regulation of SUCROSE PHOSPHATE SYNTHASE (SPS) and NR activity, both of which are key players in C/N central metabolism. Overall, it is reasonable to hypothesize that phosphorylation of F2KP and NR by an energy sensor (SnRK1) and a Ca^2+^-signal decoder (CDPKs) integrates metabolic and environmental parameters to modulate C/N balance (Fig. 1).

The modulation of F2KP and NR enzymatic activity by CDPKs may link central C/N regulatory networks to environmental circumstances through the control of metabolic enzyme activities. NR is a key enzyme in nitrate assimilation that catalyses the NAD(P)H-dependent two-electron reduction of nitrate to nitrite (Kaiser *et al.*, 2018), and its activity can be affected by CDPKs in Arabidopsis, tobacco, and spinach (*Spinacia oleracea*) (Douglas *et al.*, 1998; Lillo *et al.*, 2003; Lambeck *et al.*, 2010). A protein kinase similar to AtCPK3 in spinach is able to phosphorylate the Ser543 residue of NR (Douglas *et al.*, 1998). Lambeck *et al.* (2010) have presented evidence from *in vitro* assays that Arabidopsis AtCPK2 and AtCPK17 can phosphorylate NR in the Ser534 residue (Lambeck *et al.*, 2010). Another putative CDPK-regulated step in carbon metabolism occurs during anaplerotic replenishment of TCA intermediates. In castor bean oil seeds, RcCDPK1 phosphorylates the bacterial-type (BTPC) subunit of Class-2 PHOSPHOENOLPIRUVATE CARBOXILASE (PEPC), representing a potential link between changes in [Ca^2+^]_cyt_ and carbon fluxes to seed storage (Ying *et al.*, 2017). However, a potential link with environmental stresses to modulate seed development remains to be established.

It is well known that nitrate (NO_3_^−^) availability triggers widespread transcriptional changes, which are partly mediated by changes in [Ca^2+^] in diverse cellular compartments (Liu *et al.*, 2020). NO_3_^−^ causes specific Ca^2+^ signatures that culminate in the activation of CDPKs and CIPKs that phosphorylate channels, metabolic enzymes, and transcription factors. For instance, AtCPK10/AtCPK30/AtCPK32 phosphorylates NIN-LIKE PROTEIN (NLP) transcription factors to modulate the transcriptional responses to nitrate availability (Fig. 1). The Arabidopsis triple-mutant *cpk10 cpk30 cpk32* shows down-regulation of nitrate-responsive genes and impaired greening and expansion of the cotyledons (Liu *et al.*, 2017). Among the ~300 genes misregulated in *cpk10 cpk30 cpk32*, are ones connected to nitrate assimilation and transport, amino acid transport and metabolism, carbon/nitrogen metabolism, hormonal (cytokinin, auxin, and ABA) metabolism and signaling, nutrient transporters, proteolysis, and stress signaling. Interestingly, the basic leucine zipper *bZIP1* transcription factor is also a crucial nitrogen-signaling regulator, mediating changes in transcriptional profiles within few minutes after NO_3_^−^ supply (Para *et al.*, 2014). These quick responses are compatible with post-translational modifications in the N-responsive transcriptional machinery. bZIP1 belongs to the S-group of bZIPs that preferentially form heterodimers with C-group members, such as the sugar- and ABA-regulated transcription factor bZIP63, which has a broad impact on plant C/N metabolism (Matiolli *et al.*, 2011; Mair *et al.*, 2015). Upon phosphorylation by the energy sensor SnRK1, bZIP63 favors bZIP1 as its partner to bind to DNA. Interestingly, AtCPK3 interacts with bZIP63, suggesting a layer of regulation of nitrogen and carbon signaling that relies on Ca^2+^ signals. It is tantalizing to speculate that AtCPK3 and SnRK1 relay Ca^2+^ and energy signals to the bZIP63 phosphorylation state, thus coordinating C/N-regulated transcription in tune with the plant metabolic state and Ca^2+^-mediated environmental and developmental cues (Fig. 1). However, whether AtCPK3 directly phosphorylates bZIP63 is still an open question.

We conclude that mounting evidence suggests that Ca^2+^ signals make significant inputs into the regulation of central energy and nutrient metabolism, modulating C/N homeostasis and possibly interacting with the SnRK1–TOR energy/nutrient regulatory axis.

## Keep talking: CDPKs and SnRK1 in the organism–environment dialog

The environment is prolific in the ways that it challenges life, and plants are sessile beings that have to manage their energy and resource needs in a quick and effective manner to survive and thrive. Environmental stress can trigger a low cellular energy state and activate SnRK1, which in turn phosphorylates several metabolic enzymes and transcription factors to redirect energy and carbon fluxes. It could be suggested that stress leading to low-energy signaling through SnRK1 enables a quick repression of energy expenditure, thus saving resources through repression of growth while remobilizing reserves to fight the stress. Meanwhile, abiotic and biotic stresses trigger changes in [Ca^2+^]_cyt_ and generate signatures that correspond to specific stresses, which are decoded by the Ca^2+^-sensing toolkit that includes CIPK/CBLs and CDPKs (Dodd *et al.*, 2010). The signatures thus enable physiological and developmental decisions to be refined according to the environmental circumstances (Box 2).

Box 2. **A deep understanding of signal integration is key to improving plant fitness**

In the Tower of Babel myth, the common language of the human race was split into many different variants. The event made it difficult for people to communicate with each other and to integrate resources and workforces to reach a common goal. This could be compared with the diversity of environmental and endogenous inputs—represented by a variety of physical and chemical signals—that have to be integrated by plants to ensure survival and reproduction. Convergent signals into regulatory nodes can provide a common denominator for shared regulatory elements: a common language to activate overlapping and shared responses.

The evidence suggests that different stress signals are integrated by connected signaling networks, which use common elements—such as changing Ca^2+^ concentrations in cellular compartments or key regulatory proteins—to turn the voices in the crowd into effective crosstalk. The Ca^2+^-signaling network is thought to be capable of processing multiple incoming signals and acts together with other signaling regulators to translate environmental information into coordinated responses, a system that shows remarkable similarities to neural network computing (Dodd *et al.*, 2010). Signal integration is essential to optimize survival and reproduction because the environment presents a multitude of simultaneous challenges, which are combined with both the plant’s physiological state and developmental stage. Therefore, to elicit appropriate responses to environmental perturbations, plants need to perceive the amount of energy and resources available at a particular moment and to set the destination of these invaluable assets correctly. The following approaches could provide candidates for genetic interaction analyses between selected CDPKs and key metabolic regulators.

- Targeted yeast two-hybrid (Y2H) screening to uncover the protein–protein interaction network (PPIN) of CDPKs in model plants, deploying Y2H cDNA libraries enriched for enzymes involved in C/N metabolism
- Metabolomic analysis of single- and higher-order CDPK mutants to evaluate their impact on metabolic fluxes. These metabolomic analyses should focus on starch dynamics, sugars (including phosphorylated sugars), and amino acids and their precursors.
- Comprehensive phenotypic assessment of single- and higher-order CDPK mutants under sugar-, nitrate-, and energy-limiting conditions, for example shade, hypoxia, anoxia, and varying N supply.
- Metabolomics—and also transcriptomics and phosphoproteomics—of plants carrying mutations in CDPKs and energy/nutrient hubs (SnRK1, TOR, and HXK1), alone and in different combinations, could be employed to reveal common protein phosphorylation targets, as well as the metabolic routes affected.

These approaches could provide candidates for gene editing through CRISPR/Cas technology. Specific kinases could be edited to become constitutively active or inactive, thus achieving precise, targeted genetic manipulation to improve key agronomic traits, such as stress tolerance and productivity.

As discussed above, SnRK1 phosphorylates the sugar-responsive transcription factor bZIP63 to promote gene expression that favors catabolism (Matiolli *et al.*, 2011; Mair *et al.*, 2015), and AtCPK3 has been shown to interact with bZIP63 *in planta*, even though the significance of this interaction is still unknown. SnRK1 and AtCPK3 also converge in the phosphorylation of the Ser303 residue of AtF2KP, enabling the binding of 14-3-3 proteins (Kulma *et al.*, 2004; Nukarinen *et al.*, 2016), suggesting that the SnRK1–AtCPK3 module could converge at both the transcriptional and metabolic levels. Another clue indicating the convergence of stress and metabolic signaling through SnRK1 and CDPKs comes from rice, where the CDPK OsCPK17 plays a role in the response to cold stress (Almadanim *et al.*, 2017). This does not seem to involve the transcription regulation of known key players of the cold-stress response, suggesting that OsCPK17 may initiate an independent alternative pathway. In the initial phase of low-temperature sensing, OsCPK17 directly mediates the phosphorylation status of putative targets *in vivo*, namely OsSPS4 and the aquaporin PLASMA MEMBRANE INTRINSIC PROTEIN 2;1/6 (OsPIP2;1/2;6), and indirectly mediates the phosphorylation of OsSPS5, PYRUVATE DEHYDROGENASE E1 COMPONENT SUBUNIT ALPHA-1 (OsPDHE1-A), CHLOROPLASTIC FERREDOXIN-DEPENDENT GLUTAMATE SYNTHASE (OsFd-GOGAT), and OsNR1 (Almadanim *et al.*, 2017, 2018) (Fig. 1). Interestingly, OsCPK17 targets enzymes that are also phosphorylated by SnRK1, such as SPSs and NR, suggesting that these kinases might be involved in the integration of cold stress signaling with C/N basal metabolism to circumvent the detrimental effects of low temperatures (Halford and Hey, 2009; Almadanim *et al.*, 2017). Despite the fact that direct phosphorylation of SPSs by OsCPK17 still requires further confirmation, there is evidence of SPS phosphorylation at a similar site by a unidentified CDPK in rice that has molecular mass similar to OsCPK17 (Pagnussat *et al.*, 2002). Remarkably, most OsCPK17 targets are involved in central C/N metabolism. The OsCPK17-mediated phosphorylations that have been detected are expected to decrease the activity of most targets, as inferred from previous studies with orthologous proteins from Arabidopsis and spinach. Therefore, OsCPK17 may contribute to a metabolic adaptation mechanism through SPS, NR, or GOGAT, promoting osmotic balance through modulation of the activity of PIPs.

Interestingly, (Cotelle *et al.*, 2000) found that sugar starvation disrupts the binding of a selected set of proteins to 14-3-3 in Arabidopsis cells, including SPS and NR. The phenomenon was attributed to the loss of phosphorylation and subsequent degradation of these selected proteins. The Arabidopsis and rice SnRK1s have long been known to trigger transcriptional reprogramming upon energy-limitation induced by stressful situations such as submergence, which deprives plant cells of oxygen and limits ATP production, thus disrupting mitochondrial oxidative phosphorylation (Cho *et al.*, 2016). Taken together, these findings emphasize the relevance of the phosphorylation states of metabolic enzymes in controlling their stability. OsCPK17 transcript levels are up-regulated in young seedlings upon submergence (Mustroph *et al.*, 2010), a stress that also stimulates SnRK1 activity (Cho *et al.*, 2016), providing further evidence of the involvement of OsCPK17 in metabolic regulation. One of the putative orthologs of OsCPK17 in Arabidopsis, AtCPK1, positively regulates senescence by increasing the transcriptional activity of the ORE1 transcription factor, a master regulator of senescence (Durian *et al.*, 2020). Senescence has a crucial role in redistributing nutrients from dying to new tissues, both under stressful conditions and during the reproduction stage, to ensure optimal utilization of resources during the plant life cycle. For instance, *ORE1* seems to participate in the transcriptional reprogramming induced by mitochondrial retrograde signaling under anoxic conditions (Meng *et al.*, 2020). Importantly, SnRK1 also plays a significant role in remobilizing reserves to ensure energy availability and survival under anoxic conditions (Wurzinger *et al.*, 2018). AtCPK1 phosphorylates a disordered region of ORE1 at its C-terminal, activating its transcriptional activity but not binding of ORE1 to the DNA (Durian *et al.*, 2020). Interestingly, AtCPK1 interacts with the SnRK1 catalytic subunit KIN10 in a split-ubiquitin assay (Chen *et al.*, 2012), suggesting that AtCPK1 could participate in a broad regulatory scheme involving ORE1 and SnRK1 to cope with stress by modulating senescence and general energy metabolism, but this hypothesis remains to be tested

Ramon *et al.*, (2019) have shown that the SnRK1 catalytic subunit is excluded from the nucleus by the membrane-bound SnRK1 regulatory subunits AKINβ1 and β2, which thus prevents SnRK1 activation of target gene expression under energy-limiting conditions. This suggests that the interaction with other membrane-bound proteins could regulate SnRK1 subcellular localization. A tantalizing and speculative hypothesis is that CDPKs could phosphorylate the α or β SnRK1 subunits to release the catalytic α-subunit from the membrane, thus ‘priming’ the response to environmental or endogenous signals conveyed by changes in [Ca^2+^]_cyt_. This could be an elegant mechanism to adjust physiological outcomes by integrating stress signals with the cellular metabolic status (Fig. 1).

## Concluding remarks

Carbon fixation is the crucial biochemical process that sustains life on Earth. Plants store energy from sunlight in sugars, which are consumed to sustain basal metabolism, growth, and development. Plants live in an ever-changing environment, presenting challenges such as extreme changes in temperature, drought, scarcity of nutrients, and attacks by other organisms. To grow, plants must very tightly coordinate carbon and nitrogen levels, as well as those of inorganic phosphate and micronutrients, while managing to avoid or escape the myriad of surrounding stresses. CDPKs localize in the very core of the plant signaling networks that coordinate growth and defense. Their unique ability to directly translate the universal language of changes in cellular Ca^2+^ concentrations into target phosphorylation, together with the diversification of their gene family and their ubiquitous expression throughout plant development, make this versatile signaling relay suitable for coupling multi-signal molecular and cellular events.

**Fig. 2.**
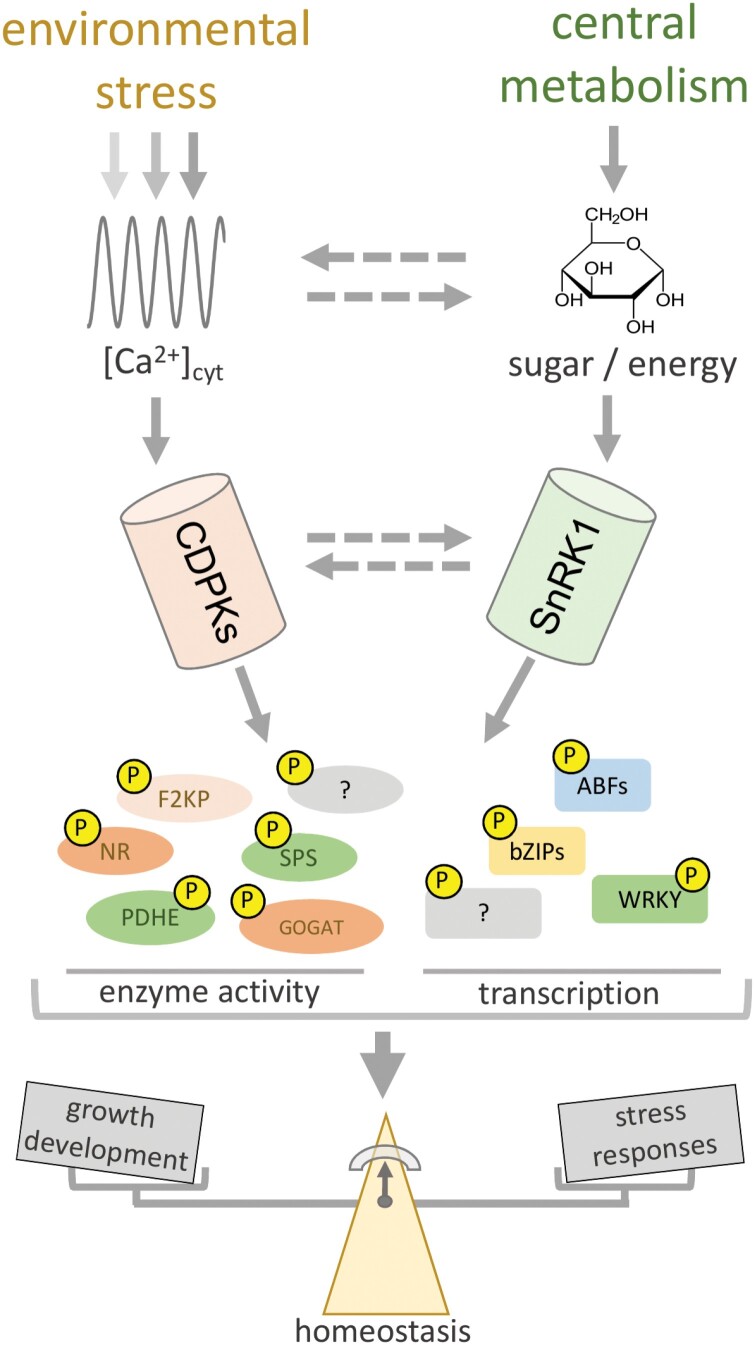
A representative model for the integration of environmental and metabolic signals by calcium-dependent protein kinases (CDPKs) and SUCROSE NON-FERMENTING RELATED KINASE1 (SnRK1). Environmental stresses trigger transient increases in cytosolic Ca^2+^ concentrations [Ca^2+^]_cyt_, which are perceived and transduced by CDPKs through phosphorylation of enzymes and transcription factors linked to C/N metabolism and stress responses. SnRK1 perceives changes in energy and sugar status, reprogramming metabolism and transcriptional events to ensure survival and allocation of resources to cope with stress. The solid arrows indicate signaling pathways supported by evidence, while the dashed arrows indicate putative connections that need further investigation. The metabolic enzymes F2KP, SPS, and NR are targeted by both CDPKs and SnRK1, suggesting that they are direct convergency nodes of the stress and sugar/energy signaling pathways. The changes in metabolic and transcriptional activities triggered by phosphorylation are thought to modulate the balance between growth and stress responses, thus maintaining plant homeostasis in challenging environments.

Here, we suggest that CDPKs are key hubs in the plant signaling pathways that link the rapid stress-responsive cellular processes with the longer-term overall regulation of metabolism and growth (Fig. 2). These kinases serve to integrate a wide variety of signals: they can be activated by phytohormones and by the perception of stress. The integration of these complex signals is crucial for activating proper networks that can coordinate, both locally and systemically, the stress-responsive mechanisms. They may also coordinate the link between stress and metabolic regulation, ultimately controlling phenotypic plasticity and reproduction. We now believe that CDPKs are key players in the signaling events discussed in this review. Despite recent progress in determining how plant defense against stress connects to the SnRK1–TOR regulatory module (Margalha *et al.*, 2019), the molecular mechanisms involved in the crosstalk that manages energy and nutrient availability in conjunction with balances between growth and defense are for the most part unknown.

The ongoing scenario of climate change enforces the need to develop biotechnological tools that can help plants to thrive under environmental constraints. Ideally, SnRK1 would be useful for enhancing crop resilience under energy-depletion and abiotic-stress conditions; however, genetic manipulation of TOR/SnRK1 has frequently resulted in deleterious and/or lethal effects (Baena-González *et al.*, 2007; Xiong *et al.*, 2013). The interplay between SnRK1 and TOR influences growth and defense, but these proteins are insufficient to fully explain growth outcomes, specifically under abiotic stress (Margalha *et al.*, 2019). Uncovering the effects of other, as yet undiscovered actors in growth–defense decisions will allow a better understanding of these mechanisms. We consider that elucidation of the tissue- and stress-specific actions of CDPKs is fundamental for the development of tools to sustain crop yields when environmental constraints impact on plant growth and production. Genetic approaches for constitutive expression or altered kinase activities of specific CDPKs may upgrade the initial stress response of the plant. Coupled to possible coordination with the SnRK1–TOR growth axis, CDPK-modified plants would possess upgraded proteomic machinery to perceive stress quickly, and to adjust cellular and metabolic activities to improve—or at least maintain—yield and productivity under stress (Box 2).
